# Additive Effects of Millimeter Waves and 2-Deoxyglucose Co-Exposure on the Human Keratinocyte Transcriptome

**DOI:** 10.1371/journal.pone.0160810

**Published:** 2016-08-16

**Authors:** Yonis Soubere Mahamoud, Meziane Aite, Catherine Martin, Maxim Zhadobov, Ronan Sauleau, Yves Le Dréan, Denis Habauzit

**Affiliations:** 1 Institut national de la santé et de la recherche médicale (Inserm), Institut de recherche en santé, environnement et travail (Irset – Inserm UMR 1085), Transcription, Environment and Cancer group (TREC), Rennes, France; 2 University of Rennes 1, Rennes, France; 3 Institute of Electronics and Telecommunications of Rennes (IETR), UMR CNRS 6164, Rennes, France; 4 University of Djibouti, Djibouti City, Djibouti; University of Alabama at Birmingham, UNITED STATES

## Abstract

Millimeter Waves (MMW) will be used in the next-generation of high-speed wireless technologies, especially in future Ultra-Broadband small cells in 5G cellular networks. Therefore, their biocompatibilities must be evaluated prior to their massive deployment. Using a microarray-based approach, we analyzed modifications to the whole genome of a human keratinocyte model that was exposed at 60.4 GHz-MMW at an incident power density (IPD) of 20 mW/cm^2^ for 3 hours in athermic conditions. No keratinocyte transcriptome modifications were observed. We tested the effects of MMWs on cell metabolism by co-treating MMW-exposed cells with a glycolysis inhibitor, 2-deoxyglucose (2dG, 20 mM for 3 hours), and whole genome expression was evaluated along with the ATP content. We found that the 2dG treatment decreased the cellular ATP content and induced a high modification in the transcriptome (632 coding genes). The affected genes were associated with transcriptional repression, cellular communication and endoplasmic reticulum homeostasis. The MMW/2dG co-treatment did not alter the keratinocyte ATP content, but it did slightly alter the transcriptome, which reflected the capacity of MMW to interfere with the bioenergetic stress response. The RT-PCR-based validation confirmed 6 MMW-sensitive genes (*SOCS3*, *SPRY2*, *TRIB1*, *FAM46A*, *CSRNP1* and *PPP1R15A*) during the 2dG treatment. These 6 genes encoded transcription factors or inhibitors of cytokine pathways, which raised questions regarding the potential impact of long-term or chronic MMW exposure on metabolically stressed cells.

## Introduction

Industrial development and new technologies have generated new anthropogenic risks for humans that include chemical risks (i.e., endocrine disrupters, nanoparticles) and physical risks (i.e., radioactivity, exposure to electromagnetic fields). The radiofrequencies range from 30 kHz to 300 GHz and have numerous applications in telecommunication (i.e., cellular networks, Local Area Networks, surveillance / radars systems) and medical systems (i.e., MRI, radiofrequency cancer ablation). Radiofrequencies have been classified into the 2B group as “possibly carcinogenic to humans” by the International Agency for Research on Cancer (IARC) based on epidemiologic studies [1]. To date, this classification remains unconfirmed by reproducible experimental studies and molecular mechanisms.

Saturation of the lower part of the microwave spectrum and a constant demand for higher data rates (for gaming, data streaming, etc.) anytime and anywhere have made new frequencies necessary. Millimeter waves (MMW, 30–300 GHz) constitute a promising new range of frequencies that may be heavily used in the coming years. The 60-GHz band is extremely attractive for high-speed wireless systems because a broadband, unlicensed bandwidth is available worldwide. High data-rate small cells and the point-to-point backhaul links of future 5G heterogeneous networks are expected to be massively deployed after 2020, which will lead to the exposure of personnel and the general public to new signals. MMW have also been used for therapeutic purposes in Eastern European countries [2,3], which raises questions regarding possible interactions between these waves and living systems. Several publications have shown both the hypoalgesic [4,5] and immune/inflammatory [6–8] effects of MMW that primarily depend on the frequency of interest. However, the mechanisms associated with these therapeutic effects remain unknown.

MMW molecular targets are unidentified to date, and no mechanisms have linked MMW with cancer or medical applications. We aimed to identify these potential links. To this end, we explored the possibility of energy stress induction. Ordinarily, alterations in cellular energy levels are observed in cancer cells. This metabolic change is characterized by a decreased aerobic mitochondrial production of ATP, which is associated with increased anaerobic glycolysis and lactic acid production [9,10]. This observation is known as the Warburg effect [11,12]. We investigated the ability of MMW to modify the cellular response to a metabolic stressor that mimicked the Warburg effect. Based on this hypothesis, we disrupted the glucose pathway by treating cells to a competitive inhibitor of glucose known as 2-deoxyglucose (2dG). This treatment decreases ATP production and starts to be used for several cancer therapeutic purposes [13,14] by inducing tumor cell apoptosis [15], as well as for tumor imaging while complexed with radioactive component [16].

Because the MMW penetration depth in tissues and liquids is small (approximately 0.5 mm at 60 GHz), the primary MMW targets are the skin (mainly keratinocytes, melanocytes, and nerves endings) and the cornea [17,18]. Because the skin is a sensitive organ that responds to various environmental insults, it represents a notably attractive target for MMW-mediated biological effects. Importantly, multiple publications have shown that the skin can function as a neuroendocrine organ that affects the global homeostasis of the endocrine and/or immune systems [19–21]. Therefore, the first aim of this study was to evaluate the effect of a 60-GHz-at-20 mW/cm^2^ treatment on a primary keratinocyte culture. The second aim was to observe the biological effects of these waves when the cells were exposed to a metabolic stressor that mimicked the Warburg effect.

## Materials and Methods

### Cell culture

Because the primary MMW target comprises the superficial layer of the skin, experiments were performed using the predominant cell type in the skin-keratinocytes. The keratinocytes were derived from two sources. The first model, primary human neonatal keratinocytes (provided by Invitrogen, Cergy Pontoise, France), was maintained in Keratinocyte-SFM medium (Gibco, Carlsbad, CA) supplemented with antibiotics (Invitrogen, Cergy Pontoise, France) and was seeded onto collagen IV-coated plates (Becton Dikinson Franklin Lakes, NJ), according to the manufacturer’s instructions. To maintain cell culture homogeneity, primary keratinocyte cultures were used between passages 4 and 8 for all experiments. For exposure experiments, 6-well collagen IV plates (Becton Dikinson Franklin Lakes, NJ) were seeded with 250 000 cells per well two days prior to the exposures. The HaCaT cell line was chosen as the second keratinocyte model and was cultured as previously described [22]. Cells (200 000 cells/well) were transferred to 6-well plates one day prior to the MMW exposure. The primary keratinocyte culture and HaCaT cell line were exposed [Expo] or sham-exposed [Sham] at 60.4 GHz in the presence or absence of 20 mM 2-deoxyglucose (2dG, Sigma-Aldrich, Saint-Quentin Fallavier, France), using a previously described exposure system [23]. The exposure protocol is summarized in Table 1.

**Table 1:** Summary of exposure protocol for the microarray experiment.

	Sham	Expo	Sham_2dG	Expo_2dG
**60-GHz exposure (20 mW/cm**^**2**^**)**	**-**	**+**	**-**	**+**
**2-deoxyGlucose (2dG) (20 mM)**	**-**	**-**	**+**	**+**
**Treatment time**	3 h	3 h	3 h	3 h
**Average Temperature in culture medium (°C)**	35.81±0.16	35.16±0.58	35.69±0.22	34.54±0.95
**Number of replicates used for microarray experiments**	4	4	4	4
**Number of replicates used for RT-PCR validation**	5	5	6	6

### ATP content measurement

HaCaT cells were trypsinized and harvested by centrifugation after the treatments. Cells were lysed, and the ATP concentration was quantified using the Roche ATP bioluminescence Assay kit, according to the manufacturer’s instructions (Roche diagnostics, Meylan, France). The sample bioluminescence was measured at 562 nm with a VERITAS microplate luminometer (Turner BioSystems, Sunnyvale, CA, USA).

### Exposure system and experimental setup

The 6-well culture plate was placed in the MEMMERT UE400^™^ incubator and exposed from the bottom by a standard pyramidal horn antenna. Cells were exposed [Expo] or Sham-exposed [Sham] with an average Incident Power Density (IPD) of 20 mW/cm^2^ for 3 hours. This IPD level was the limit defined by the International Commission on Non-Ionizing Radiation Protection (ICNIRP) guideline [24] for frequencies with spatial maximum power density averages that not exceed 1 cm^2^. To protect cells from MMW exposure-induced overheating, the temperature increase was compensated by decreasing the incubator set point. The temperature in the cell medium was monitored using a 4-channel Reflex fiber optic thermometer (NEOPTIX, Quebec, Canada). Complete biological replicates were performed (n = 4 for HaCaT; n = 5 to 6 for primary keratinocytes–Table 1), and the order of each manipulation was randomly determined.

### Microarray

Primary keratinocytes were immediately harvested following exposure. Total RNA was purified using the Qiagen RNeasy kit (Qiagen, Hilden, Germany) and quantified using the Nanodrop 1000 spectrophotometer (Nanodrop Technology, Cambridge, UK), as previously described [25].

A one-color whole gene expression modification analysis was performed using the Agilent Whole Human Genome 8x60K Microarray Kit (Agilent Technologies, Les Ulis, France). Four replicates per condition were analyzed with the GeneSpring GX software (Agilent Technologies, Les Ulis, France). Briefly, the expression profile was log2-transformed and normalized (scaling and baseline transformation), and 23 767 gene entities were eventually detected on the microarrays. Expression level-based filters (intensity greater than 125) with standard deviations < 0.5 were used for probe selection as previously described [25]. The complete data set was deposited in the Gene Expression Omnibus (GEO) database (www.ncbi.nlm.nih.gov/geo, GEO series accession number: GSE83829).

### Microarray analysis

Gene expression modifications were compared using a two-tailed Mann-Whitney test. A microarray analysis was performed using multiple Mann-Whitney tests (detailed in S1 Fig) for confirmation. P-values were adjusted by controlling the false discovery rate (FDR) with the Benjamini & Hochberg (BH) correction for multiple testing. A gene was considered significantly differentially expressed if the adjusted p-value was below 0.05, and the absolute fold-change (FC) was above 2 or 1.5. Four direct side-by-side comparisons (S1 Fig) were performed. The first aimed to determine the MMW effect alone (Sham *versus* Expo). The second characterized the 2dG effect on gene expression (Sham *versus* Sham_2dG). The third aimed to determine if the co-exposure altered gene expression (Sham_2dG *versus* Expo_2dG). The fourth was an analysis of the global MMW effect on gene expression apart from the 2dG treatments ((Sham+Sham_2dG) *versus* (Expo+Expo_2dG)). For the last comparison, the two Sham conditions and the two Expo conditions were pooled.

### RT-PCR validation

Five hundred nanograms of RNA were reverse-transcripted using the M-MLV Reverse Transcriptase kit, according to the manufacturer’s instructions (Invitrogen, Carlsbad, CA). Primers were designed using QuantPrime (www.quantprime.de), verified on Primer-blast (www.ncbi.nlm.nih.gov/tools/primer-blast/), and purchased from Sigma Aldrich (Sigma Aldrich, St. Louis, MO). The primer sequences are presented in Table 2. Two housekeeping genes, Glyceraldehyde-3-Phosphate Dehydrogenase (GAPDH) and TATA Box-Binding Protein (TBP), were used to normalize gene expression. PCR products, amplified with the IQ SYBR Green supermix (Bio-rad, Hercules, CA), were continuously measured using the CFX Connect^™^ Real-Time PCR Detection System (Bio-Rad, Hercules, CA). All data were incorporated into the CFX Manager gene analysis software (Bio-Rad), and the relative changes in gene expression were analyzed using the delta Ct method [26]. The RT-PCR-based validation was performed using mRNA obtained from the four mRNA preparations used in the microarray experiments along with one or two additional mRNAs, depending on the condition being tested (see materials and methods, Table 1). The results were statistically analyzed using the two-tailed Wilcoxon-Mann-Whitney test. Each value was provided as the mean with its standard deviation (SD) and was considered statistically significant when p < 0.05. Statistics were calculated with R from the BiostaTGV interface http://marne.u707.jussieu.fr/biostatgv/.

**Table 2:** List of primers used to validate the differentially expressed genes determined by the GeneSpring analysis.

Gene Symbol	Forward	Reverse	Refseq #
CSRNP1	ATAGGATCTGCGACCCTGAGAC	TGTGTGGTCCATCTGGCACTTG	NM_033027
FAM46A	ACACCACTCACGCTCAAGGAAG	TCCATCGGTCAGAGTCATTGCAC	NM_017633
HBEGF	GAGACTTGTGCTCAAGGAATCGG	CCTCTGCAGTCTGAAATCACCTTG	NM_001945
HS3ST1	AGCAAGTATGGAAGCGGGACTC	AGTTGCTGCACACGTAGCCATC	NM_005114
LIF	TGCTTCATCCGGCTTAGCTTGG	AGTTTGTCTTTCTCGAAGCCCATC	NM_002309
PPP1R15A	GAGGAGGCTGAAGACAGTGG	AATTGACTTCCCTGCCCTCT	NM_014330
TRIB1	CTTCTGGTTGGACGATACCC	TTCCAAGACGGACTCAAACC	NM_025195
SOCS3	TCGCCTTAAATGCTCCCTGTCC	TCCAGGCTGAGTATGTGGCTTTC	NM_003955
IER3	GAACTGCGGCAAAGTAGGAG	AACTTACGACCCACCACCAG	NM_003897
SPRY2	CGCAGAAAGAAGAGAATCCAAGGG	GAACACATCTGAACTCCGTGATCG	NM_005842
GAPDH	TGCACCACCAACTGCTTAGC	GGCATGGACTGTGGTCATGAG	NM_002046
TBP	TGCACAGGAGCCAAGAGTGAA	CACATCACAGCTCCCCACCA	NM_003194

## Results

### Treatment with 2dG, but not with MMW, affects the intracellular ATP concentration

**Fig 1:**
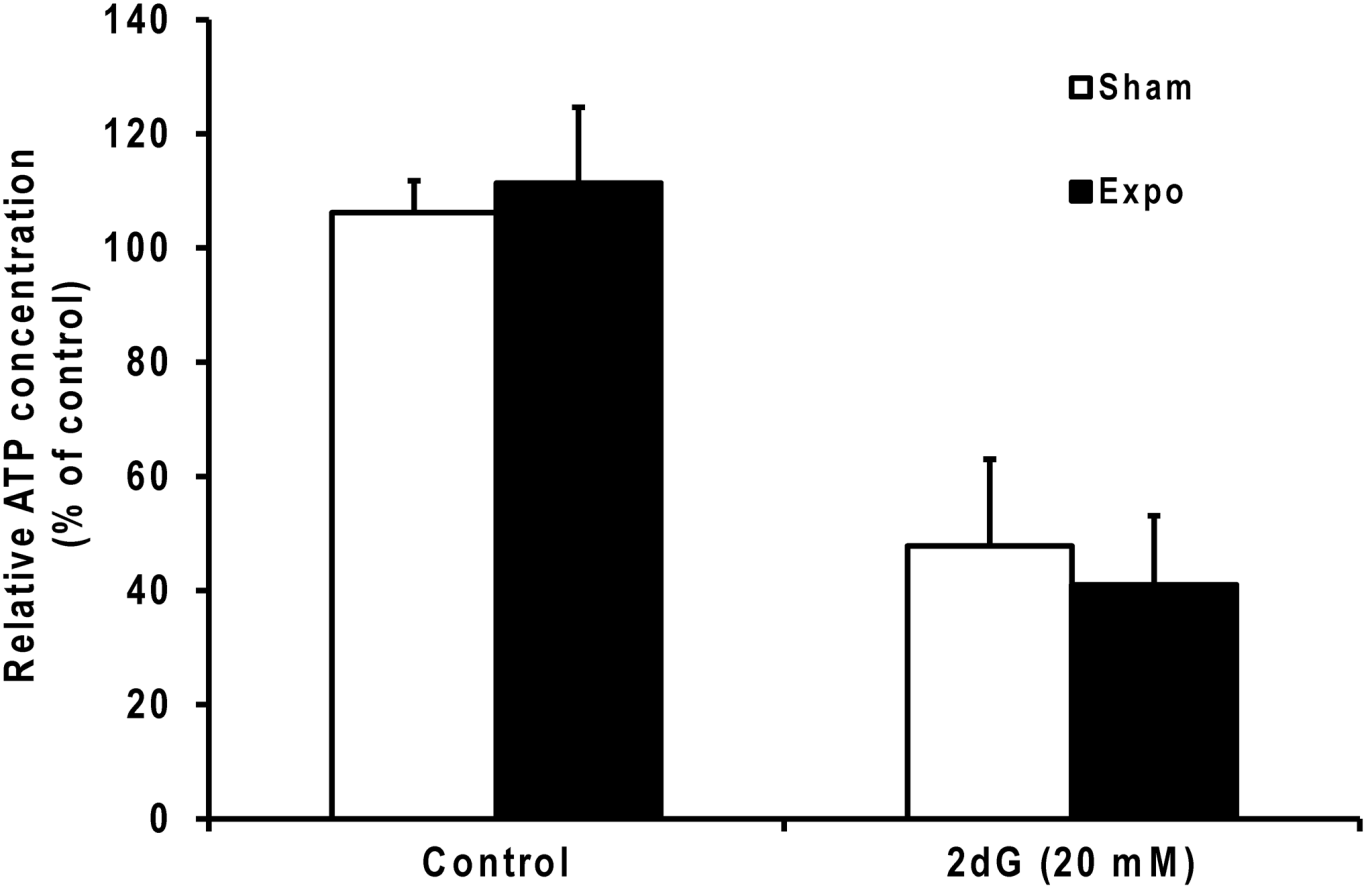
Relative cellular ATP concentration. HaCaT cells were treated with or without 20 mM 2dG for 3 h. Additionally, cells were exposed (black histograms) or unexposed (white histograms) to 60.4 GHz (20 mW/cm^2^). Data are presented as the mean of four independent experiments and are expressed as the percent of an independent unexposed control that was set to 100%.

The human HaCaT keratinocyte cell line was treated with 20 mM 2dG and/or exposed to 60.4 GHz MMW with an IPD of 20 mW/cm for 3 hours. The MMW did not significantly modify the ATP level after a 3-h exposure at 60.4 GHz (Fig 1). However, the 2dG treatment induced a significant 2-fold decrease in the ATP contents of unexposed and MMW-exposed cells. Moreover, no significant difference in the ATP level was observed with MMW co-exposure during the 2dG treatment. These data clearly indicate that MMW does not potentiate the 2dG-triggered energetic stress.

### Treatment with 2-deoxyglucose, but not with MMW, dramatically affects the human keratinocyte transcriptome

Human primary keratinocytes were treated with or without 20 mM 2dG and exposed [Expo] or unexposed [Sham] to an average Incident Power Density (IPD) of 20 mW/cm^2^ for 3 hours. Four exposure conditions were tested (Sham, Expo, Sham_2dG and Expo_2dG; Table 1). First, the MMW effect on the primary keratinocyte transcriptome was evaluated. Without the 2dG treatment, a Mann-Whitney test comparison of the [Sham] and [Expo] conditions (Table 1, S1 Fig) showed that the primary keratinocyte transcriptome was unchanged (Table 3). This result demonstrated that in athermic conditions without co-exposure, acute MMW stimulation did not stress cells enough to alter gene expression, which confirmed our previously published data [25].

**Table 3:** Conditions compared and genes determined as statistically relevant by Mann-Whitney analyses.

Conditions compared	BH	FC	BH corrected p-value	BH uncorrected p-value	Gene list
Sham *versus* Expo	**Yes**	**0**	**Yes**	**0**	
	**No**	**0**	**No**	**0**	
Sham_2dG *versus* Expo_2dG	**Yes**	2.0	0.039	0.021	CSRNP1
		1.73	0.039	0.021	FAM46A
		2.74	0.039	0.021	HBEGF
		2.29	0.039	0.021	HS3ST1
		1.78	0.039	0.021	LIF
		1.67	0.039	0.021	TRIB1
	**No**	1.52		0.021	ALDH1L1
		1.50		0.043	DUSP5
		1.66		0.043	IER3
		1.54		0.021	PIM1
		1.61		0.043	ERRFI1
		1.60		0.043	PPP1R15A
					Down:
		1.55		0.021	ZNF555
		1.57		0.021	HUWE1
(Sham+Sham_2dG) *versus* (Expo +Expo_2dG)	**Yes**	1.78	0.013	0.005	SPRY2
		1.51	0.013	0.009	IER3
	**No**	1.79		0.009	SOCS3
		1.63		0.046	CSRNP1
		1.58		0.046	HES1
					Down:
		1.78		0.0063	ZNF441
		1.60		0.036	LOC100130876

The 2dG effect on keratinocyte gene expression was also evaluated. Using a side-by-side Benjamini Hochberg-corrected Mann-Whitney test, the 2dG_Sham and Sham conditions were compared using a cut-off FC value of 2, where 665 differentially expressed probes were identified, or 1.5, where 770 differentially expressed probes were identified. Among those in the last list (FC > 1.5 and a BH-corrected p-value < 0.05), 632 probes corresponded to unique coding genes, with 286 probes upregulated and 388 downregulated by the 2dG treatment. The absolute FC values fell between 1.5 and 30, and their average was 2.6 (S1 Table).

Functional enrichments were performed on the 632 coding genes using the DAVID software (David version 6.7; http://david.abcc.ncifcrf.gov/). Biological categories were considered enriched when the Bonferroni-corrected p-value fell below 0.05. The 2dG-affected biological processes and molecular functions were mainly associated with DNA-binding activity and transcription and were strongly associated with transcriptional repressor activity (S2–S5 Tables). A KEGG (Kyoto Encyclopedia of Genes and Genomes) pathways database analysis through the WEBGESTALT toolkit (WEB-based GEne SeT AnaLysis Toolkit [27]) was performed (S6 Table). The main enrichment was associated with the p53 signaling pathway, which was consistent with a potential role for 2dG in cell cycle arrest. We also highlighted the enrichment of the neurotrophin signaling pathway with an emphasis on the *NFκBIE* gene, which is implicated in cell survival. Moreover, 2dG interferes with cellular communication by increasing the potential cytokine-cytokine receptor interaction (i.e., with *IL6*, *IL20*, *IL24 and CXCL3*) and factors involved in MAPK signaling pathways (i.e., *DUSP10*, *DDIT3*, *GADD45A and GADD45B*). In the KEGG-enriched pathway, the most remarkable effect involved protein processing in the endoplasmic reticulum (i.e., *PPP1R15A*, *DDIT3*, *XBP1*, *HSPA1B and EIF2AK3*). This highlighted an effect on endoplasmic reticulum stress, which is specifically characterized by a high overexpression of the DNA-Damage-Inducible Transcript 3 (*DDIT3*), which is a dominant-negative inhibitor of CCAAT/enhancer-binding protein (C/EBP).

### MMW effects on the human keratinocyte transcriptome with the 2dG co-treatment

Two Mann-Whitney statistical analyses were performed. These analyses aimed to determine if MMW modified the primary keratinocyte transcriptome during the 2dG co-treatment (Table 1, S1 Fig). Our objective was to determine the MMW effect on the 2dG-stressed keratinocytes [sham_2dG] *versus* [Expo_2dG] by performing a Mann-Whitney test (Table 3, S1 Fig). The analysis with the BH correction showed that six genes were differentially expressed with fold changes above 1.5; fourteen genes were identified without the BH correction (the gene are detailed in Table 3).

A second Mann-Whitney test was performed to compare the expo and sham conditions regardless of the 2dG treatment (Table 3; S1 Fig). This analysis was performed to determine if a general, 2dG-independent MMW effect was evident on the keratinocyte transcriptome ([Sham+Sham_2dG] *versus* [Expo+Expo_2dG]). With the BH correction, this test identified two genes as differentially expressed with fold changes above 1.5; seven genes were observed without the BH correction. Most of these genes were also observed in the previously detailed analysis (Table 3).

Ten genes were selected for validation by RT-PCR based on these analyses. This gene list contained all of the genes revealed by the Mann-Whitney analysis with the BH correction (8 genes) and two from the Mann-Whitney test without the BH correction that showed low p-values. Altogether, the ten genes selected from the group of nineteen for the RT-qPCR-based validation were: heparin-binding EGF-like growth factor (*HBEGF*), cysteine and serine-rich nuclear protein 1 (*CSRNP1*), leukemia inhibitory factor (*LIF*), heparan sulfate (glucosamine) 3-O-sulfotransferase 1 (*HS3ST1*), family with sequence similarity 46, member A (*FAM46A*), tribbles pseudokinase 1 (*TRIB1*), sprouty homolog 2 (*SPRY2*), immediate early response 3 (*IER3*), protein phosphatase 1, regulatory subunit 15A (*PPP1R15A*) and suppressor of cytokine signaling 3 (*SOCS3*), which belongs to the same protein family as *SPRY2* (Table 3).

### Gene validation by RT-qPCR

**Fig 2:**
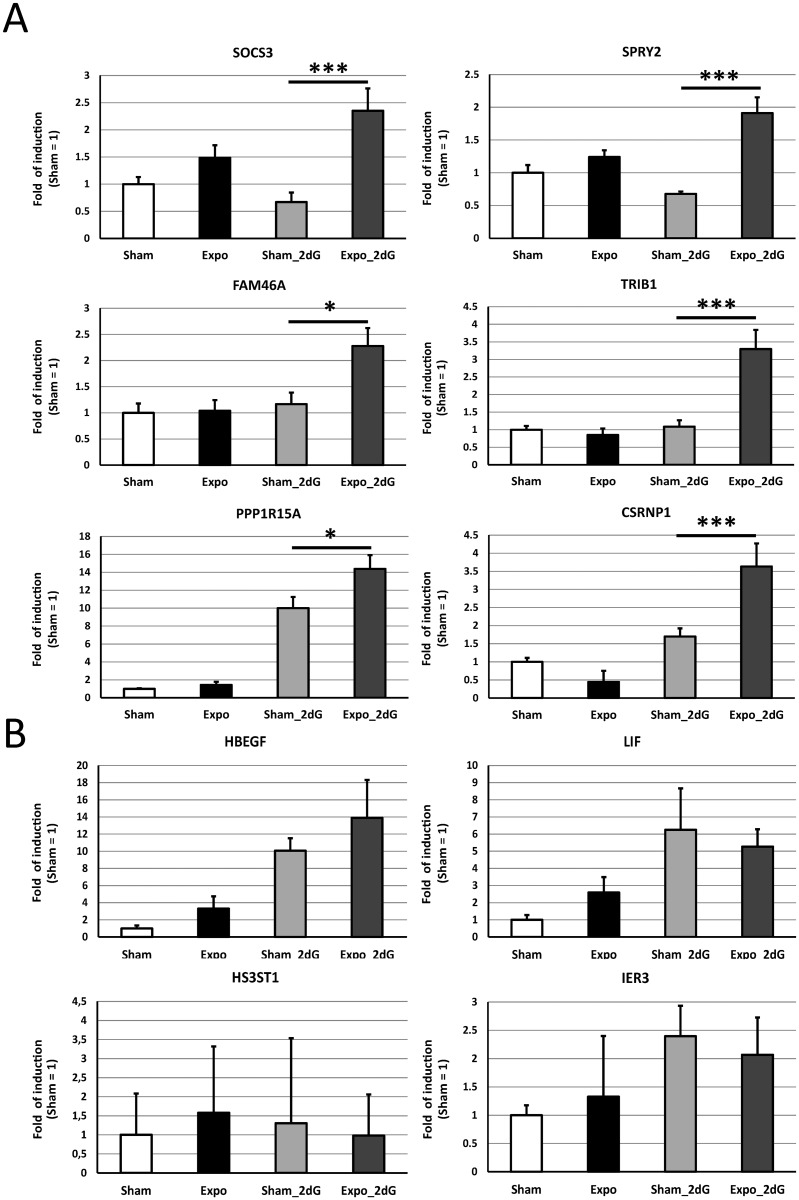
The gene expression profiles of the validated genes. Histograms represent the RT-qPCR data. The expression profiles were classified depending on their validation profiles as follows: (A) validated by RT-PCR or (B) not validated by RT-PCR.

Six genes were validated as MMW sensitive (Fig 2A). Of the four unconfirmed genes, one (*HS3ST1*) was not validated due to the high instability of its Ct values, and the profiles of three genes (*IER3*, *LIF* and *HBEGF*) were inconsistent with those determined by microarray (Fig 2B). The six validated genes were *SOCS3*, *SPRY2*, *FAM46A*, *TRIB1*, *PPP1R15A* and *CSRNP1* (Fig 2). For these genes, a relevant MMW effect was observed when MMW was applied in combination with 2dG. Altogether, these PCR validations have permit to validate the existence of six genes differentially expressed while response to metabolic stroke was associated with acute MMW exposure.

## Discussion

We observed no effects of the MMW treatment alone on the human primary keratinocyte transcriptome in athermic conditions, and MMW did not alter the ATP contents of exposed cells. The 2dG treatment affected ATP metabolism, which altered gene expression. The most notable response was the decrease in gene expression, which was consistent with adaptation to metabolic stress. It is noteworthy that the 2dG treatment increased the expression of genes implicated in transcriptional repression. Moreover, the treatment did not activate genes involved in cell apoptosis, which was consistent with the literature [28]. Therefore, the cells survived during the 3-hour treatment by activating pro-survival phosphorylation cascades [29]. This observation was consistent with the KEGG-enriched MAPK signaling pathway.

Interestingly, the 2dG treatment triggered a stress response known as the unfolded protein response (UPR) by affecting several relevant genes [30,31]. Among the ER-localized UPR early stress sensors, only eukaryotic translation initiation factor 2 alpha kinase 3 (*EIF2AK3*, also known as *PERK*) was upregulated, with a fold change of 2.16. A specific *EIF2AK3* increase without a modification to *IRE1* has been shown to be a cellular marker of adaptation and survival during hypoxia [32]. The *XBP1* gene was also upregulated by this treatment (FC = 1.95), along with its target gene, *DNAJB9* (upregulated, FC = 4.5). The target genes of the *EIF2AK3* activity cascade [33,34], such as *DDIT3* (also known as *CHOP* or *GADD153*, FC = 28) and *PPP1R15A* (also known as *GADD34*, FC = 5.6) were also upregulated. Several reports addressing glucose starvation have highlighted *DDIT3* and *PPP1R15A* as affected genes, and *DDIT3* has been described as a promoter of apoptosis under conditions of ER stress [35,36]. Because the 2dG treatment was short (3 h), the 2dG treatment induced increases in genes associated with cell rescue, such as *DNAJB9* (also known as *ERDJ4*, FC = 4.5), but not its associated genes, such as *HSPA5* (also known as *GRP78 or BIP*) and *HYOU1* (also known as *ORP150*). The absence of *HSPA5* and *HYOU1* upregulation was noteworthy because these genes were previously reported as upregulated by 2dG [37,38]. This discrepancy may be due to our 3-hour 2dG treatment, which may have focused on the early response genes of the cell. Had the treatment been prolonged, more apoptosis-associated genes would have been observed.

However, the 2dG treatment activated cytokine production by increasing *IL6* (FC = 7) and *IL6R* (FC = 2) expression, along with *CXCL3* (FC = 11). Two cytokines that are specific to keratinocytes [39] and implicated in T cell-mediated skin inflammation [40] were also differentially expressed (*IL20* and *IL24*, FC = 5.3 and 5.6, respectively). These pro-inflammatory effects activate the immune system, particularly neutrophils [41,42]. Therefore, the immune response is expected to sustain the positive effects of the 2dG treatments against cancer when it is used as a co-treatment [16].

Regarding the MMW effect on keratinocytes, we found that exposure did not modify the cellular ATP cell content. This observation indicated that acute MMW exposure did not to target mitochondrial function. Moreover, the profile of differentially expressed genes observed after the MMW exposure was not caused by increased metabolic stress but instead reflected the capacity of MMW to interfere with the cellular response when cells were treated with 2dG. The microarray analysis highlighted that acute MMW exposure alone had no impact on gene expression, which was consistent with a previous study [25]. Even if the 2dG treatment induced endoplasmic reticulum (ER) stress, the MMW co-treatment did not modify ER stress as previously described [22]. However, the statistical analyses showed additive effects on gene expression only when cell homeostasis was disturbed by 2dG. Of these potential biomarkers, ten genes were selected for a RT-PCR-based validation, and six of the ten selected genes were validated (*SPRY2*, *SOCS3*, *PPP1R15A*, *FAM46A*, *TRIB1* and *CSRNP1)*. This demonstrated a clear and relevant MMW effect. Five of the six validated genes (*SOCS3*, *SPRY2*, *PPP1R15A*, *TRIB1* and *CSRNP1*) were associated with the cell surface receptor signaling pathway (GO:0007166; Biological process). This involvement suggests that MMW co-exposure may modify the cell surface. Moreover, four of these genes (*SOCS3*, *SPRY2*, *PPP1R15A*, *TRIB1*) were also involved in negatively regulating signaling (GO:0023057), particularly signal transduction (GO:0009968).

MMW exposure directly increased the expression of *SPRY2* and *SOC3*. *Sprouty homolog 2* (*SPRY2*) inhibits receptor tyrosine kinases (RTKs) through the ERK pathway [43]. This gene is essential for development and the prevention of diseases, such as cancer and leukemia. It is noteworthy that *SPRY2* is connected to the RAS/RAF signaling pathway that was previously observed to be modulated after electromagnetic exposure [44,45]. The *suppressor of cytokine signaling 3* (*SOCS3*) is a STAT-induced STAT inhibitor that tempers the effects of several cytokines and specifically targets the JAK2/STAT5 and JAK2/STAT3 pathways. *SOCS3* was not previously observed as a specific target of the millimeter waves, but modulation of the STAT pathway has been previously reported [46–48]. Together, the up-regulation of both *SPRY2* and *SOCS3* suggests that the cytokine cascades that target RAS/RAF and JAK/STAT are inhibited after a short MMW stimulation. *SOCS3*, *PIM1*, *TRIB1*, *PPP1R15A*, *CSRNP1 (also named AXUD1) and IER3* have also been associated with thrombocythemia and have been precisely linked to the JAK/STAT pathway [49]. Interestingly, the JAK/STAT signaling pathway is activated by the IL6/IL6R signaling pathway, which is upregulated by 2dG. Therefore, *SOCS3*, *SPRY2*, *PPP1R15A*, *TRIB1* and *CSRNP1* are involved in cellular communication. This last association implies a need for further investigations into the risks of long-term, chronic MMW exposure on humans.

## Conclusions

Treatment with 2dG induced a strong modification of the gene expression profile, but the MMW effect on gene expression was weaker. No genes were modified when the cells were exposed to MMW alone under athermic conditions. When cells were co-treated with MMW and 2dG, only six genes were identified and validated by RT-qPCR. Of these validated genes, *SPRY2* and *SOCS3* constituted an early response in which cytokine pathways could be inhibited, and *CSRNP1* corresponded to a transcription factor. Because these upregulated genes can initiate a late transcriptome modification in the exposed cells, they may be involved in a long-term response. Together, the differentially expressed genes indicated activity through the JAK/STAT signaling pathway. In the future, investigations into the effects of long-term, chronic MMW exposure will be necessary to understand the implications of these cytokine and transcription factor modifications.

## Supporting Information

S1 Fig Comparative strategies for Mann-Whitney tests. Four direct side-by-side comparisons were performed. The results of these comparisons are detailed in Table 3.
(DOC)

S1 Table List of the 626 differentially expressed coding genes with FC values > 1.5. Genes selected from Mann-Whitney tests.
(XLS)

S2 Table Summary of the genes implicated in GO terms. (XLS)

S3 Table Statistically relevant GO terms implicated in Biological Process (BP). (XLS)

S4 Table Statistically relevant GO terms implicated in Molecular Function (MF). (XLS)

S5 Table Statistically relevant GO terms implicated in Cellular Component (CC). (XLS)

S6 Table Enriched KEGG pathways classified according to their p-Values. (XLS)
