# Supplementary material for: Additive Effects of Millimeter Waves and 2-Deoxyglucose Co-Exposure on the Human Keratinocyte Transcriptome
Source: PLoS One. 2016 Aug 16;11(8):e0160810. doi: 10.1371/journal.pone.0160810 (PMC4986955; doi:10.1371/journal.pone.0160810)
Supplement: S1 Fig — Four direct side-by-side comparisons were performed. The results of these comparisons are detailed in Table 3. (DOC) [file pone.0160810.s001.doc]

**Supplementary material**

ADDITIVE EFFECTS OF MILLIMETER WAVES AND 2-DEOXYGLUCOSE COEXPOSURE ON THE HUMAN KERATINOCYTE TRANSCRIPTOME

Yonis Soubere Mahamoud, Meziane Aite, Catherine Martin, Maxim Zhadobov, Ronan Sauleau, Yves Le Dréan & Denis Habauzit

**Legends of supplementary tables**

**S1 Table. List of the 626 differentially expressed coding genes with FC values > 1.5. Genes selected from Mann-Whitney tests.**

**S2 Table. Summary of the genes implicated in GO terms**

**S3 Table. Statistically relevant GO terms implicated in Biological Process (BP)**

**S4 Table. Statistically relevant GO terms implicated in Molecular Function (MF)**

**S5 Table. Statistically relevant GO terms implicated in Cellular Component (CC)**

**S6 Table. Enriched KEGG pathways classified according to their p-Values**

**Table of contents**

**S1 Fig. Comparative strategies for Mann-Whitney tests. Four direct side-by-side comparisons were performed. The results of these comparisons are detailed in Table 3.**


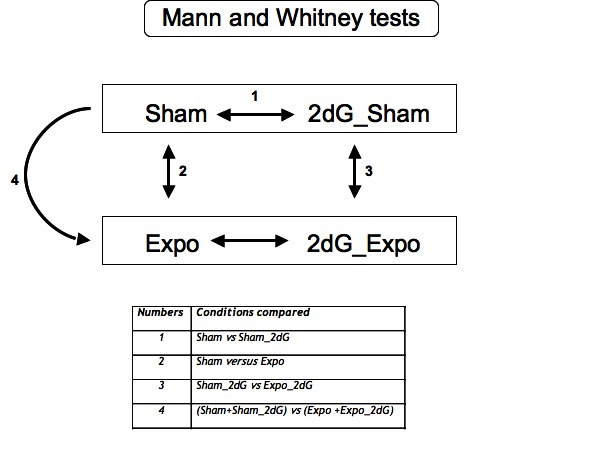


**S1 Fig. Comparative strategies for Mann-Whitney tests. Four direct side-by-side comparisons were performed. The results of these comparisons are detailed in Table 3.**
